# Characterizing the Epithelial Transcriptome of Type‐2 High Chronic Rhinosinusitis

**DOI:** 10.1002/alr.70235

**Published:** 2026-08-09

**Authors:** Brian Cameron, Jeffanie Gayoso, Christina Dorismond, Ping Li, Mason Krysinski, Rakesh Chandra, Justin Turner, Naweed Chowdhury

**Affiliations:** ^1^ Department of Otolaryngology—Head and Neck Surgery Vanderbilt University Medical Center Nashville Tennessee USA; ^2^ Department of Otolaryngology—Head and Neck Surgery University of Mississippi Medical Center Jackson Mississippi USA; ^3^ Department of Otolaryngology—Head and Neck Surgery University of Alabama at Birmingham Birmingham Alabama USA

**Keywords:** chronic rhinosinusitis, epithelium, gene expression, RNA‐sequencing, transcriptomics

## 1 Introduction

1 **Key Point**

• Bulk RNA‐sequencing of middle meatus brushings from 84 CRS patients identified 18 differentially expressed genes (11 upregulated, 7 downregulated) in Type‐2 high versus Type‐2 low CRS.
• Top overexpressed genes included CST1, PTHLH, CLC, FETUB, ITLN1, and POSTN, reflecting eosinophil infiltration, extracellular matrix remodeling, and goblet cell biology, while downregulated genes, including KRT14 and SCGB3A1 suggest impaired basal cell homeostasis and immunoregulation.
• These epithelial‐derived transcriptomic markers may support disease stratification and identification of candidates for biologic therapy in CRS.

Recently, our understanding of the pathophysiology of chronic rhinosinusitis (CRS) has progressed considerably. CRS is now understood to result largely from aberrant inflammatory processes involving a mixture of Type 1 (T1), Type 2 (T2), and Type 3 (T3) inflammatory pathways, each with associated immune cells and biomarkers, leading to putative endotypes of disease [1, 2]. In Western populations, CRS with nasal polyps (CRSwNP) is characterized by T2 inflammation and is associated with elevated T2 cytokines (IL‐4, IL‐5, and IL‐13) and tissue eosinophilia. This finding has led to endotype‐based treatments, like monoclonal antibodies targeting the T2 inflammatory cascade, which have been shown to significantly improve symptoms and outcomes in treatment‐refractory patients [3]. Prior studies have examined gene expression in nasal polyp tissue, identifying T2 and non‐T2 endotypes with DEGs including CLC, CPA3, and CCL26 [4], and nasal brushing microarray data from the SINUS‐52 trial have identified transcriptional clusters associated with dupilumab response [5]. However, bulk RNA‐sequencing of the epithelial surface in cytokine‐defined T2‐high versus T2‐low CRS has not been previously reported. With this study, we aimed to characterize the differential gene expression between T2‐high and T2‐low CRS to identify genes and pathways that may serve as candidate biomarkers for disease stratification.

## 2 Methods

Patients undergoing endoscopic sinus surgery (ESS) for CRS were enrolled in a prospective longitudinal observational study and followed for a minimum of 6 months. At the beginning of surgery, middle meatal brushings were used to collect superficial nasal epithelial cells. RNA was then extracted and processed as previously described [6]. A multiplex flow cytometric bead assay was used to quantify IL‐5 cytokine concentrations from concurrently collected nasal mucus samples from the middle meatus to define a T2 high‐inflammatory state.

Bulk transcriptional reads mapping to the human genome (hg38) were isolated and used for subsequent expression analysis using the limma package, with differentially expressed genes (DEGs) visualized using volcano plots. Gene‐set enrichment analysis (GSEA) was conducted using clusterProfiler with the WikiPathways collection reference. Due to statistically significant differences in age, race, and asthma prevalence between groups (Table 1), the DEG and GSEA models were adjusted for these factors. Detailed laboratory and statistical methods are included in the supplement.

**TABLE 1:** Demographic and clinical characteristics of the study cohort by Type 2 inflammatory status.

	Type 2 high/low			
Characteristic	High *N* = 42^a^	Low *N* = 42^a^	*p*‐value^b^	Overall *N* = 84^a^
Age (years)	43.3 (15.1) [18.0–81.0]	53.8 (15.2) [22.0–78.0]	0.002	48.6 (16.0) [18.0–81.0]
Sex			0.28	
Female	18.0 (42.9%)	23.0 (54.8%)		41.0 (48.8%)
Male	24.0 (57.1%)	19.0 (45.2%)		43.0 (51.2%)
Race			0.022	
Caucasian	31.0 (77.5%)	39.0 (92.9%)		70.0 (85.4%)
African American	6.0 (15.0%)	0.0 (0.0%)		6.0 (7.3%)
Hispanic	1.0 (2.5%)	1.0 (2.4%)		2.0 (2.4%)
Asian	0.0 (0.0%)	1.0 (2.4%)		1.0 (1.2%)
Other	1.0 (2.5%)	1.0 (2.4%)		2.0 (2.4%)
Unknown	1.0 (2.5%)	0.0 (0.0%)		1.0 (1.2%)
Body mass index (kg/m^2^)	26.8 (3.7) [17.8–35.9]	28.7 (6.7) [16.7–55.1]	0.39	27.8 (5.5) [16.7–55.1]
Asthma			0.016	
No	16.0 (38.1%)	27.0 (64.3%)		43.0 (51.2%)
Yes	26.0 (61.9%)	15.0 (35.7%)		41.0 (48.8%)
Allergic rhinitis			0.15	
No	9.0 (21.4%)	15.0 (35.7%)		24.0 (28.6%)
Yes	33.0 (78.6%)	27.0 (64.3%)		60.0 (71.4%)
Preoperative Lund–Mackay CT score	14.6 (5.5) [3.0–24.0]	12.0 (5.0) [3.0–22.0]	0.053	13.3 (5.4) [3.0–24.0]

^a^ Mean (SD) [Min—Max]; *n* (%).

^b^ Wilcoxon rank sum test; Pearson's chi‐squared test; Fisher's exact test.

## 3 Results

Overall, 84 CRS patients undergoing ESS were included (Table 1). After adjustment for age, race, and asthma, differential expression analysis identified 18 DEGs between T2‐high and T2‐low CRS, with 11 upregulated and 7 downregulated (Figure 1).

**FIGURE 1:**
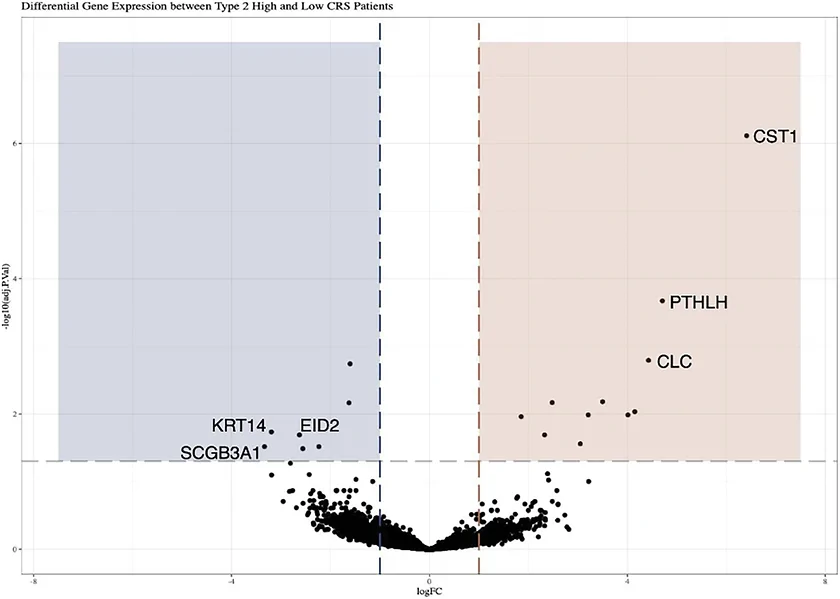
Volcano plot of differentially expressed genes between Type 2 high and Type 2 low CRS. Dashed vertical lines indicate the logFC threshold (±1.5), and the dashed horizontal line represents the adjusted *p*‐value significance threshold (*p*_adj_ < 0.05). The shaded blue region (left) highlights significantly downregulated genes; the shaded red region (right) highlights significantly upregulated genes in Type 2 high CRS. Labeled genes represent the top three upregulated and top three downregulated DEGs by absolute log fold change: CST1 (cystatin SN; logFC = 6.41, *p*_adj_ < 0.001), PTHLH (parathyroid hormone‐like hormone; logFC = 4.71, *p*_adj_ < 0.001), CLC (Charcot–Leyden crystal protein/galectin‐10; logFC = 4.43, *p*_adj_ = 0.002), SCGB3A1 (secretoglobin family 3A member 1; logFC = −3.34, *p*_adj_ = 0.030), KRT14 (keratin‐14; logFC = −3.19, *p*_adj_ = 0.018), and EID2 (EP300‐interacting inhibitor of differentiation 2; logFC = −2.63, *p*_adj_ = 0.020).

Among the upregulated genes, cystatin (CST1) demonstrated the largest fold change (logFC = 6.41; 95% CI, 4.70–8.11; *p*_adj_ < 0.001), followed by parathyroid hormone‐like hormone (PTHLH; logFC = 4.71; 95% CI, 3.18–6.23; *p*_adj_ < 0.001), Charcot–Leyden crystal protein (CLC; logFC = 4.43; 95% CI, 2.85–6.00; *p*_adj_ = 0.002), fetuin B (FETUB; logFC = 4.15; 95% CI, 2.48–5.81; *p*_adj_ = 0.009), intelectin‐1 (ITLN1; logFC = 4.01; 95% CI, 2.37–5.65; *p*_adj_ = 0.010), and periostin (POSTN; logFC = 3.50; 95% CI 2.14–4.85; *p*_adj_ = 0.007). Seven genes were significantly downregulated, including secretoglobin 3A1 (SCGB3A1; logFC = −3.34; 95% CI, −4.83 to −1.84; *p*_adj_ = 0.030) and keratin‐14 (KRT14; logFC = −3.19; 95% CI, −4.56 to −1.82; *p*_adj_ = 0.018). The complete list of DEGs is provided in Table S1 and Figure S1. GSEA revealed enrichment of G‐protein coupled receptor pathways containing T2‐associated chemokine receptors (CCR3, PTGDR2) and hematopoietic differentiation pathways in T2‐high CRS, with downregulation of cell cycle regulation, ciliary biology, and proteasomal degradation pathways (Table S2 and Table S3).

## 4 Discussion

In this study, we used an untargeted transcriptomic approach to identify genes and pathways associated with T2‐high CRS. CST1 and CLC demonstrated markedly elevated expression, consistent with eosinophil infiltration and concordant with prior whole‐transcriptome analyses of nasal polyp tissue that identified these genes among the top DEGs in the T2 endotype [4]. Notably, CLC was also the only gene to remain differentially expressed after covariate adjustment in a related analysis of patients prescribed dupilumab for surgically refractory CRSwNP, supporting its role as a marker of T2‐driven disease [6]. POSTN, a matricellular protein implicated in fibroblast differentiation and polyp formation, was also significantly overexpressed [7], consistent with its established role in more severe CRSwNP. ITLN1, a lectin involved in innate mucosal defense, also showed increased expression, consistent with IL‐13‐driven ITLN1 induction in T2‐high airway epithelium and mucus pathology [8]. PTHLH has also been associated with glandular hyperplasia in CRS, and is primarily localized to serous cells within the nasal epithelium [9]. FETUB encodes for a plasma protein thought to play a role in inflammatory progression of respiratory diseases, which was previously found to be elevated in patients with CRS [5].

Our finding of low KRT14 expression suggests impaired basal cell renewal, but the evidence in CRS is conflicting, as it was found to be elevated in another study [9], though this work did not stratify by endotype. Underexpression of secretoglobulin genes has also been noted in other chronic respiratory diseases, such as asthma and COPD, and may reflect loss of anti‐inflammatory and immunoregulatory function in the epithelium [10]. Taken together, downregulation of these pathways may promote ongoing inflammation and prevent epithelial restoration, leading to persistent activation of the T2 inflammatory cascade.

Limitations include the utilization of bulk RNA‐sequencing, which captures a heterogeneous cell population rather than cell‐type‐specific expression; modest sample size given the diversity of known CRS endotypes; and the use of brushings, which do not sufficiently sample deeper stromal and inflammatory cell populations, but provide a less invasive method for collection that is better suited for serial sampling. Despite these limitations, the data in this exploratory study provide insights into epithelial cell behavior in T2‐dominant CRS, which can inform our understanding of CRS pathogenesis.

## 5 Conclusions

T2‐high CRS is associated with an epithelial transcriptomic profile characterized by overexpression of eosinophil and extracellular matrix remodeling genes, alongside reduced expression of basal cell homeostasis markers and immunoregulatory pathways. These findings may support early stratification of disease severity and identification of candidates for biologic therapy using transcriptomics.

## Funding

This project was supported by the Burroughs Wellcome Fund PSIA award (N.I.C.), NIAID L30AI186158 (N.I.C.), NIH R01 AG065550 (J.H.T.), K24 AI177947 (J.H.T.), and CTSA award UL1TR000445 from the National Center for Advancing Translational Sciences. Its contents are solely the responsibility of the authors and do not necessarily represent official views of the National Center for Advancing Translational Sciences or the National Institutes of Health. Justin Turner has received grant support from the NIH/National Institute of Allergy and Infectious Diseases (NIAID) and NIH/National Institute on Aging (NIA). Naweed Chowdhury has received grant support from the Burroughs Wellcome Fund, the NIH/National Cancer Institute, and NIH/NIAID.

## Conflicts of Interest

Rakesh Chandra is a consultant for Sanofi, Regeneron, Optinose, and Lyra Therapeutics. Justin Turner has received personal fees from Regeneron. Christina Dorismond is a consultant for Myelin Healthcare. The other authors declare no conflicts of interest.

## Supporting information




**Supporting File 1**: alr70235‐sup‐0001‐FigureS1.png. **Supplementary Figure S1** – Heatmap of top 50 differentially expressed genes in type 2 high vs low CRS.

**Supporting File 2**: alr70235‐sup‐0002‐SuppMat.docx
