# Supplementary material for: Characterizing the Epithelial Transcriptome of Type‐2 High Chronic Rhinosinusitis
Source: Int Forum Allergy Rhinol. 2026 Aug 9;16(10):1206–9. doi: 10.1002/alr.70235 (PMC13634546; doi:10.1002/alr.70235)
Supplement: Supplementary file 1 [file ALR-16-1206-s002.docx]

**Characterizing the Epithelial Transcriptome of Type-2 High Chronic Rhinosinusitis**

Supplemental materials

**Supplementary Methods**

*Recruitment and Inclusion/Exclusion Criteria*

Adult patients presenting to Vanderbilt University Medical Center for endoscopic sinus surgery (ESS) for a clinical diagnosis of chronic rhinosinusitis (CRS) were eligible for inclusion based on EPOS^S1^ or ICAR:RS^S2^ criteria after failing a trial of appropriate medical management including topical nasal corticosteroids, at least one course of oral antibiotics and steroids, and nasal saline irrigations. Patients were excluded if they had odontogenic rhinosinusitis, mycetoma, known autoimmune or granulomatous disorder, cystic fibrosis, or recent systemic steroid use (within 4 weeks of surgery). Tissue eosinophil counts were obtained from review of hematoxylin and eosin-stained histopathology slides by a board-certified pathologist, with the average count over 5 high power fields used as the representative value. The study was reviewed and approved by the Vanderbilt University Medical Center Institutional Review Board (IRB number 121467).

*Type 2 Mucus Cytokine Quantification*

Prior to starting ESS, two 9 × 24 mm polyurethane sponges (Summit Medical, St Paul, MN) were placed into the middle meatus of each subject under direct endoscopic visualization and left in place for 5 minutes to absorb nasal mucus. Sponges were then centrifuged at 14,000 × g for 10 minutes to elute mucus, vortexed, and the supernatants were separated and frozen at −80°C for pooled analysis. Cytokine concentrations were subsequently quantified using a multiplex cytokine bead assay (BD Biosciences, Franklin Lakes, NJ), with data analyzed using BD FCAP Array v3.0 software. Type 2 high inflammatory status was defined using the median value of IL-5 across the study cohort as a threshold, with patients at or above the median classified as type 2 high and those below classified as type 2 low, consistent with prior cytokine-based endotyping methods used at our institution.^S3^

*Cell Collection and Sequencing Methods*

At the beginning of surgery, a cytology brush was used to collect superficial cell samples from the middle meatus bilaterally under endoscopic guidance and placed into a tube containing 500µL of OMNIgene ORAL (DNA Genotek), which was frozen at −80°C and stored. Samples were later thawed and vortexed for 2 minutes, with 250µL of sample used for total RNA extraction. Briefly, the aliquot was homogenized in 600µL QIAzol (Qiagen) and 500µL of 2.0 mm zirconium oxide beads (Next Advance) using a Bullet Blender homogenizer, maintaining temperatures at or near 4°C using the dry ice cooling system. The sample was then treated with 100µL of genomic DNA Eliminator solution (Qiagen) to remove genomic DNA and 180µL of chloroform was added for phase separation. The total RNA in the aqueous phase was purified using the RNeasy Mini spin columns as recommended by the Qiagen RNeasy protocol. RNA integrity and quantification were assessed using an Agilent Bioanalyzer RNA 6000 Nano/Pico Chip (Agilent Technologies).

Illumina sequencing libraries were prepared using the NEBNext Ultra II RNA Library Prep Kit (NEB #E7775), with library quality assessment using an Agilent Bioanalyzer DNA High Sensitivity chip. Sequencing was performed on an Illumina NovaSeq6000 platform with 2×150 base pair reads, with a sequencing depth of approximately 40 million paired-end reads per sample. Adapter removal and quality-based trimming of the raw reads were performed using Trimmomatic with default parameters.^S4^ Trimmed reads shorter than 50nt were discarded. FastQC was used before and after trimming to assess read quality.^S5^

*Differential Expression Analysis and Statistical Methods*

The preprocessed reads were mapped to a reference transcriptome index generated from the hg38 genome and quantified using kallisto.^S6^ Samples with fewer than 1 million pseudoaligned reads were filtered. The feature counts were combined into a single data frame using a custom R script. Differential expression analysis was then performed by comparing type 2 high versus type 2 low groups using the limma package.^S7^ Genes with an absolute value log_2_ fold change > 1.5 with an adjusted p-value < 0.05 were treated as differentially expressed. Gene-set enrichment analysis (GSEA) for human gene sets in the WikiPathways reference database^S8^ was performed using clusterProfiler,^S9^ with a false discovery rate of 0.05 used to determine statistical significance.

Due to statistically significant differences in age (p = 0.002), race (p = 0.022), and asthma prevalence (p = 0.016) between type 2 high and type 2 low groups (Table 1), both the DEG and GSEA models were adjusted for these covariates. Between-group differences were compared using the Wilcoxon rank sum test for continuous variables or Pearson’s Chi-squared test and Fisher’s exact test for categorical variables, with an alpha level of 0.05 for statistical significance.

**Supplementary References**

S1. Fokkens WJ, Lund VJ, Hopkins C, et al. European position paper on rhinosinusitis and nasal polyps 2020. Rhinology. 2020;58(Suppl S29):1-464.

S2. Orlandi RR, Kingdom TT, Smith TL, et al. International consensus statement on allergy and rhinology: rhinosinusitis 2021. Int Forum Allergy Rhinol. 2021;11(3):213-739.

S3. Turner JH, Chandra RK, Li P, et al. Identification of clinically relevant chronic rhinosinusitis endotypes using cluster analysis of mucus cytokines. J Allergy Clin Immunol. 2018;141(5):1895-1897.e7.

S4. Bolger AM, Lohse M, Usadel B. Trimmomatic: a flexible trimmer for Illumina sequence data. Bioinformatics. 2014;30(15):2114-2120.

S5. Andrews S. FastQC: a quality control tool for high throughput sequence data. Available at: http://www.bioinformatics.babraham.ac.uk/projects/fastqc/. Accessed 2024.

S6. Bray NL, Pimentel H, Melsted P, Pachter L. Near-optimal probabilistic RNA-seq quantification. Nat Biotechnol. 2016;34(5):525-527.

S7. Ritchie ME, Phipson B, Wu D, et al. limma powers differential expression analyses for RNA-sequencing and microarray studies. Nucleic Acids Res. 2015;43(7):e47.

S8. Martens M, Stumpf A, Kuber K, et al. WikiPathways: connecting communities. Nucleic Acids Res. 2021;49(D1):D613-D621.

S9. Wu T, Hu E, Xu S, et al. clusterProfiler 4.0: a universal enrichment tool for interpreting omics data. Innovation (Camb). 2021;2(3):100141.

**Table S1.** Differentially Expressed Genes in Type 2 High Versus Type 2 Low CRS After Adjustment for Age, Race, and Asthma. Genes are separated into upregulated (A) and downregulated (B) panels and sorted by descending absolute log fold change. logFC = log_2_ fold change; p_adj_ = Benjamini-Hochberg adjusted p-value.

**A. Upregulated Genes in Type 2 High CRS**

| **Gene** | **logFC** | ***p*_adj_** |
| --- | --- | --- |
| CST1 | 6.41 | < 0.001 |
| PTHLH | 4.71 | < 0.001 |
| CLC | 4.43 | 0.002 |
| FETUB | 4.15 | 0.009 |
| ITLN1 | 4.01 | 0.010 |
| POSTN | 3.50 | 0.007 |
| NTRK1 | 3.21 | 0.010 |
| TRPM5 | 3.05 | 0.028 |
| KCNJ16 | 2.48 | 0.007 |
| CST2 | 2.33 | 0.020 |
| CDH26 | 1.85 | 0.011 |

**B. Downregulated Genes in Type 2 High CRS**

| **Gene** | **logFC** | ***p*_adj_** |
| --- | --- | --- |
| SCGB3A1 | −3.34 | 0.030 |
| KRT14 | −3.19 | 0.018 |
| EID2 | −2.63 | 0.020 |
| DCTPP1 | −2.56 | 0.033 |
| RHOQP1 | −2.24 | 0.030 |
| ALDH2 | −1.63 | 0.007 |
| ALDH3A1 | −1.60 | 0.002 |

**Supplementary Table S2:** WikiPathways GSEA results for significantly upregulated pathways in type 2 high CRS

| **Pathway** | **NES** | **adj p val** | **Genes** |
| --- | --- | --- | --- |
| HEMATOPOIETIC_STEM_CELL_DIFFERENTIATION | 2.10 | 0.002 | TPO, GATA2, CBFA2T3, NFE2, KCNH2, CSF1, ITGA2B, LYL1, CXCR4, RUNX1, THRB, GYPA, IL1A, GATA1, FOSB, TRAF3IP3, IL3, FLI1, IKZF1, IL5, CD34, NCKAP1L, SPI1, FOS, KLF1, RHOH |
| MARKERS_OF_KIDNEY_CELL_LINEAGE | 2.06 | 0.004 | KDR, SFRP1, EGLN3, SALL1, TNC, GDNF, SMAD5, FAT4, KIT, NTN1, WNT4, ALDH1A2, TBX18, EYA1, EMX2 |
| PEPTIDE_GPCRS | 2.02 | 0.002 | CCR3, TACR1, FPR1, CXCR2, MC2R, CXCR4, TRHR, NPY6R, TAC4, GALR1, SSTR2, CCR4, OPRK1, HCRTR1, CCR9, CCR1, CXCR1, FPR2 |
| GPCRS_CLASS_A_RHODOPSINLIKE | 1.87 | < 0.001 | CCR3, P2RY14, FFAR3, CYSLTR2, HCAR3, PTGDR2, HRH2, FPR1, CXCR2, ADRA2A, GPR161, MC2R, P2RY13, ADRA1A, GPR65, ACKR3, HTR2B, SUCNR1, CXCR4, DRD2, NMUR2, CNR1, ADORA3, TRHR, GPR18, OR10A4, NPY6R, GALR1, FFAR2, GPR42, SSTR2, F2RL3, OR2A4, P2RY10, CCR4, OPRK1, P2RY6, GPR173, NMUR1, DRD1, HCRTR1, ADORA1, CCR9, CCR1, CHRM1, CXCR1, FPR2 |

NES = normalized enrichment score; adj p val = P values adjusted using the Benjamini-Hochberg method for multiple comparisons with false discovery rate of 0.05. GSEA performed on expression counts adjusted for statistically significant differences in age, race, and asthma.

**Supplementary Table S3:** WikiPathways GSEA results for significantly downregulated pathways in type 2 high CRS

| **Pathway** | **NES** | **adj p val** | **Genes** |
| --- | --- | --- | --- |
| G1_TO_S_CELL_CYCLE_CONTROL | -2.01 | < 0.001 | CDK7, CREB1, MNAT1, CCNE2, E2F3, CREB3, MCM6, PRIM1, WEE1, CDKN1B, CDKN1C, ORC2, MCM5, ATF6B, CCNB1, POLA2, TFDP1, MDM2, RB1, CCNA1, MCM3, POLE2, ORC3, CCND1, RPA3, CDC45, CDK1, ORC5, MYC, CDKN2B, CDKN2A, PCNA |
| RETINOBLASTOMA_GENE_IN_CANCER | -1.93 | < 0.001 | CDC7, CDK4, SKP2, SMC3, MAPK13, TYMS, POLD3, CCDC6, CCNE2, E2F3, PRMT2, RFC3, TTK, SIN3A, TOP2A, ZNF655, MCM6, RRM1, SMC1A, PRIM1, WEE1, CDKN1B, CHEK1, RBP1, SAP30, DCK, MSH6, CCNB2, CCNB1, TFDP1, MDM2, RB1, BARD1, KIF4A, SUV39H1, MCM3, POLE2, DHFR, CCND1, SMC2, RPA3, CDC45, CDC25B, CDK1, MYC, CCNA2, PCNA |
| PROTEASOME_DEGRADATION | -1.93 | < 0.001 | PSMA5, UBA7, HLA-A, PSMD6, RPN2, PSMB4, PSMB7, PSMA2, PSMA3, UBA1, PSMA7, PSMA6, PSMD11, PSMB2, PSMB3, PSMB5, PSMD5, PSME3, PSMD13, IFNG, PSMD3, PSMC1, PSMC2, PSMB6, PSMB9, PSMD8, UCHL1, UBE2D1, PSMB8 |
| INTRAFLAGELLAR_TRANSPORT_PROTEINS_BINDING_TO_DYNEIN | -1.91 | 0.006 | IFT80, DYNC1LI2, IFT140, DYNC2LI1, IFT81, DYNLRB1, IFT46, DYNLL2, IFT22, DYNLL1, DYNLT3 |
| CILIOPATHIES | -1.89 | < 0.001 | ARL3, SPATA7, AHI1, NEK2, TMEM216, TTBK2, USP9X, BBS5, TMEM107, SPAG1, TCTN3, GAS8, HYLS1, IFT43, CSPP1, RP2, XPNPEP3, CEP120, CFAP53, TCTN2, CFAP52, GALNT11, DNAI1, MKKS, TRAF3IP1, ATXN10, AK7, IFT80, MCIDAS, PIBF1, PIK3R4, RSPH1, CEP78, TTC8, CEP19, SUFU, RAB23, BBIP1, PDE6D, IFT140, TCTN1, RSPH4A, DYNC2LI1, IFT81, EVC, ARL2BP, MAK, OCRL, TMEM138, BBS4, DNAL1, B9D1, CCNO, KIAA0586, TOPORS, INPP5E, DNAAF5, BBS2, OFD1, CCDC65, LZTFL1, NEK8, NME9, NPHP1, DNAAF2, ARL6, IFT57, DCDC2, ZMYND10, B9D2, DRC1, FAM161A, ARL13B, BBS12, CCDC103, TMEM231 |
| PPARALPHA_PATHWAY | -1.89 | 0.009 | RXRA, APOA2, CYP4A11, SCP2, CCND1, CDK1, MYC, ACAA1, EHHADH, PLTP |
| GLYCINE_METABOLISM_INCLUDING_IMDS | -1.89 | 0.010 | IBA57, LIPT2, LIAS, NFU1, LIPT1, SHMT1, BOLA3, ISCU, HSCB, GCSH, PNPO |
| CILIARY_LANDSCAPE | -1.88 | < 0.001 | IFT122, IFT172, LCN2, WNK1, ANKS6, ARMC8, TFAP2A, RANBP9, BBS7, DCAF11, HDAC1, HDAC2, MKS1, RANBP10, SNAP29, GID4, IFT27, IFT88, MYL6B, TIPRL, CDH23, DNPEP, COPS4, RAB8A, IFT52, EXOC6B, LSM4, MAEA, MFAP1, MCM10, TMED1, VAPB, COPS7A, EXOC5, MCM8, YPEL5, PGRMC2, SPATA7, CNOT6L, NFKB1, RALB, EXOSC9, GLA, RAB2A, DGKE, PSMD12, BBS5, COPS2, ARHGDIA, COPS3, AGPAT2, IFT43, PAFAH1B1, COX6C, TFAP2E, XPNPEP3, CNOT10, EXOC2, DCAF7, MCM6, NUP133, TRAF3IP1, WEE1, ACSL3, CTNNB1, CAMK2A, IFT80, ECHS1, MCM5, NDUFA5, TTC8, AFG3L2, BBIP1, POM121, CNOT9, IFT140, RAB3IL1, RB1, COPS5, IFT81, NINL, PSMD13, APMAP, DYNLRB1, EHD3, BBS4, CALM1, MCM3, IFT46, DYNLL2, HTRA2, VPS4A, IFT22, SSNA1, TFAP2C, RPGR, DYNLL1, CD2BP2, BBS2, DVL3, LZTFL1, NUDC, NEK8, COPS6, PSMD8, DYNLT3, IFT57, IQGAP2, COPS8, MSH2, EXOSC4, ZMYND19, LRPPRC, USH1C |
| GENES_RELATED_TO_PRIMARY_CILIUM_DEVELOPMENT_BASED_ON_CRISPR | -1.88 | < 0.001 | KIF3B, TMEM216, TTBK2, BBS5, TMEM107, KIF3A, TCTN3, IFT43, CEP120, MIB1, TCTN2, MKKS, TRAF3IP1, CEP162, IFT80, PIBF1, TTC8, CEP19, IQCE, RAB23, IFT140, TCTN1, DYNC2LI1, IFT81, EVC, BBS4, B9D1, CDK20, CEP44, IFT46, INPP5E, DYNLL1, SASS6, BBS2, OFD1, LZTFL1, TULP3, ARL6, IFT57, KATNB1, ARL13B, BBS12, TMEM231 |
| OXIDATION_BY_CYTOCHROME_P450 | -1.87 | 0.002 | CYP2D6, CYP4F3, CYP1A1, CYP2F1, CYP4X1, CYP11B1, CYB5R4, CYP24A1, CYB5R2, CYP17A1, CYP4F2, CYP26C1, CYP2C18, CYP2G1P, CYP4F12, CYB5R3, CYP4A22, CYP39A1, CYP7B1, POR, CYB5B, CYP4B1, CYP4F11, CYP26B1, CYP4A11, CYP4Z1, CYP11A1, CYP51A1, CYB5A, CYP26A1, CYP2A13, CYP4F8, CYP2A7, CYP1B1, CYP19A1 |
| METHIONINE_METABOLISM_LEADING_TO_SULFUR_AMINO_ACIDS_AND_RELATED_DISORDERS | -1.86 | 0.008 | MAT1A, MTR, AHCY, CTH, GNMT, MAT2B |
| PATHWAYS_INTO_METHIONINE_AND_FOLATE_CYCLES | -1.85 | 0.006 | ALPL, SHMT1, SLC52A3, MAT1A, AOX1, DHFR, MTR, HCFC1, AHCY, GNMT, SLC52A1, FOLR1, RFK, MAT2B, PNPO |
| CELL_CYCLE | -1.82 | < 0.001 | CDK7, CCNE2, E2F3, TTK, ANAPC13, E2F4, SMAD3, ANAPC11, MCM6, SMC1A, CDC23, WEE1, RBX1, CDKN1B, CDKN1C, ORC2, CHEK1, MCM5, TGFB1, CCNB2, CUL1, CCNB1, TFDP1, MDM2, ANAPC1, CDC14A, RB1, SMC1B, YWHAE, SFN, CCNA1, MCM3, SMAD4, PTTG1, ORC3, BUB3, CCND1, CDC45, CDC25B, CDC6, CDK1, ORC5, MAD2L2, WEE2, GSK3B, MYC, CCNA2, CDKN2B, CDKN2A, PCNA |
| 3Q29_COPY_NUMBER_VARIATION_SYNDROME | -1.78 | 0.010 | PIGZ, FBXW7, PIGX, PIK3R3, CASP7, NCBP2, UBXN7, PXN, RNF168, CEP19, TGFB1, FBXO45, RABL2B, PAK2, DYNC2LI1, TM4SF19, DYNLRB1, RNF8, DYNLL2, GRIA1, DYNLL1, MAD2L1BP, NF2, DYNLT3, MYC, SLC40A1 |
| DISORDERS_OF_MITOCHONDRIAL_HOMEOSTATIS_DYNAMICS_PROTEIN_IMPORT_AND_QUALITY_CONTROL | -1.77 | 0.008 | YME1L1, AIFM1, PAM16, TXN2, USP9X, OPA3, MFN2, XPNPEP3, ATAD3A, COQ5, MFN1, SLC25A46, MIPEP, PPA2, GFER, PYCR2, PMPCA, AFG3L2, DNAJC19, UBXN6, STAT2, PINK1, HTRA2, TIMMDC1, CLPP, VCP, UBXN1, COQ3, C1QBP, MSTO1 |
| ALLOGRAFT_REJECTION | -1.76 | 0.005 | FASLG, HLA-DQA2, CSNK2A2, CTLA4, GZMB, HLA-DOA, TGFB1, HLA-DPA1, CASP8, CXCL12, COL5A1, MICA, IFNG, C2, FAS, CASP9, HLA-DPB1, C1QB, HLA-DQB1, ABCB1, STAT1, HLA-DMB, HLA-DRB1, C1QA, HLA-DMA, C1QC, CXCL9, CXCL11 |
| PARKINUBIQUITIN_PROTEASOMAL_SYSTEM_PATHWAY | -1.70 | 0.007 | CCNE1, PSMD10, CASK, HSPA8, PSMC3, SNCAIP, TUBB4B, HSPA1A, PSMD2, PSMD12, FBXW7, PSMD6, TUBB2B, UBA1, PSMD11, CUL1, TUBB3, HSPA14, GPR37, CASP8, HSPA1B, PSMD14, PSMD5, PSMD13, HSPA5, TUBA4B, PSMD3, UBE2L6, TUBA1A, PSMC1, CASP1, PSMC2, PSMD8, STUB1, TUBA8, TUBA1C |
| MIRTARGETED_GENES_IN_LYMPHOCYTES | -1.50 | 0.002 | TXN2, TYMS, MARS2, TRIP13, ARHGDIA, NELFCD, NXN, SLC12A4, COMMD9, GTPBP3, TMEM59, E2F3, ARID4B, ATG3, SLC25A22, SRSF9, ATP6V1F, GNPNAT1, PPP3R1, RAB6A, DHX40, RAD23B, SPRYD4, BRI3BP, GALNT1, PTGES3L-AARSD1, TRMT1, DCAF7, PTBP1, SHOC2, ABHD10, KPNA3, SERP1, ARFIP2, IDH1, SMC1A, ELMOD2, IGF2BP1, PRIM1, FADS1, PWP1, CARHSP1, CTNNB1, HDAC4, POLR2C, USP1, AURKB, CDKN1B, YIF1B, CDKN1C, PANX1, PTPRJ, ARFIP1, EIF4E, SCYL1, SH3BGRL3, SLC25A1, TNFRSF10A, MAGT1, RAB23, TMEM43, ZNF622, POM121, PPIB, CPNE8, NT5C3A, POLA2, CNOT9, FGF2, ANAPC1, DHX57, GFPT1, RB1, NCL, PPP3CA, CXCL12, UHRF1, GRPEL2, RAB34, WDR82, MRPL20, MYLIP, LAMC1, MPDU1, MAPK7, TUBA1A, SSNA1, MRPS33, PTMA, AKAP8, UTP15, CCND1, G6PD, ANP32B, AP3D1, LAMTOR3, TMED2, RFT1, WDFY1, MTX1, TMED10, TUSC2, CYP51A1, FADS2, MOV10, NF2, PNP, PHC2, TPPP3, WNT5A, COL4A2, KCNN4, SRPRB, BACE1, ADAR, MRC2, PLXND1, POLD2, CDIPT, C1QBP, CYP1B1, PTRH1, CDKN2A, MRPS24, FRG1, VCAM1 |
| ALZHEIMERS_DISEASE | -1.49 | 0.007 | TRAF2, TUBB4B, WNT2B, ITPR1, RELA, NFKB1, NRBF2, PSMD2, DKK2, PSMD12, PIK3R3, WNT3A, CALML4, DVL2, PIK3R1, AKT2, PSMA5, PSEN2, PSMD6, CASP7, PPP3R1, TUBB2B, PSMB4, PSMB7, WNT5B, VDAC3, EIF2S1, FADD, ATG101, PSMA2, PSMA3, EIF2AK2, LRP6, MAP2K7, SLC25A5, CTNNB1, CSNK2A2, GRIN1, PIK3R4, PSMA7, DKK1, CSNK2A1, MAPK9, PSMA6, CHRNA7, EIF2AK3, FRAT1, TUBB3, PSMB2, RYR3, MTOR, PSMB3, PSMB5, RB1CC1, DKK4, PPP3CA, CASP8, PSMD14, PSEN1, PSMD13, PPP3CC, CALM1, PSMD3, NCSTN, TUBA1A, APOE, MAP2K1, FAS, PSMC1, APH1B, PSMC2, WIPI2, CASP9, PSMB6, DVL3, ADAM17, CALML3, CHUK, SLC25A4, NOX4, PLCB1, WNT5A, PSMD8, ADRM1, CACNA1F, MAPK3, GSK3B, TUBA8, BACE1, CSNK2B, TUBA1C, CDK5, NOX1, PSENEN, CALML5 |
| ALZHEIMERS_DISEASE_AND_MIRNA_EFFECTS | -1.49 | 0.007 | TRAF2, TUBB4B, WNT2B, ITPR1, RELA, NFKB1, NRBF2, PSMD2, DKK2, PSMD12, PIK3R3, WNT3A, CALML4, DVL2, PIK3R1, AKT2, PSMA5, PSEN2, PSMD6, CASP7, PPP3R1, TUBB2B, PSMB4, PSMB7, WNT5B, VDAC3, EIF2S1, FADD, ATG101, PSMA2, PSMA3, EIF2AK2, LRP6, MAP2K7, SLC25A5, CTNNB1, CSNK2A2, GRIN1, PIK3R4, PSMA7, DKK1, CSNK2A1, MAPK9, PSMA6, CHRNA7, EIF2AK3, FRAT1, TUBB3, PSMB2, RYR3, MTOR, PSMB3, PSMB5, RB1CC1, DKK4, PPP3CA, CASP8, PSMD14, PSEN1, PSMD13, PPP3CC, CALM1, PSMD3, NCSTN, TUBA1A, APOE, MAP2K1, FAS, PSMC1, APH1B, PSMC2, WIPI2, CASP9, PSMB6, DVL3, ADAM17, CALML3, CHUK, SLC25A4, NOX4, PLCB1, WNT5A, PSMD8, ADRM1, CACNA1F, MAPK3, GSK3B, TUBA8, BACE1, CSNK2B, TUBA1C, CDK5, NOX1, PSENEN, CALML5 |

NES = normalized enrichment score; adj p val = P values adjusted using the Benjamini-Hochberg method for multiple comparisons with false discovery rate of 0.05. GSEA performed on expression counts adjusted for statistically significant differences in age, race, and asthma.

**Supplementary figure S1** – Heatmap of top 50 differentially expressed genes in type 2 high vs low CRS.
